# Effect of balance training on static and dynamic balance performance in male adolescents: role of training frequency

**DOI:** 10.1186/s13104-022-06177-y

**Published:** 2022-12-12

**Authors:** Thomas Muehlbauer, Arne Grundmann, Lukas Vortkamp, Simon Schedler

**Affiliations:** Division of Movement and Training Sciences/Biomechanics of Sport, University of Duisburg-Essen, Gladbecker Str. 182, 45141 Essen, Germany

**Keywords:** Postural control, Adolescence, Dose–response relationship

## Abstract

**Objective:**

Previous studies reported significant improvements in static and dynamic balance performance following balance training during adolescence. However, it is unclear how equal training volume but different training frequencies per week affect training-induced adaptations. Thus, the present study investigated the effects of balance training frequency (i.e., 2 × 30 min per week or 3 × 20 min per week) on measures of static and dynamic balance in healthy male adolescents.

**Results:**

Irrespective of balance training frequency, significant pretest to posttest improvements were detected for static (i.e., One-Legged Stance test, standing time duration) and dynamic (i.e., Lower Quarter Y Balance test, reach distance) balance performance. However, no group × test interactions were found. These results imply that balance training is effective to improve static and dynamic balance performance in healthy male adolescents, but the effectiveness seems unaffected by the applied balance training frequency.

## Introduction

Numerous studies have shown that balance training (BT) is an effective training regimen to improve static and dynamic balance performance in healthy adolescents [1–3]. For example, Schedler et al. [2] detected significantly increased stance durations for the One-Legged Stance test (OLS) and improved reach distances for the Lower Quarter Y Balance test (YBT-LQ) following seven weeks of BT (2 sessions/week) in male adolescents (mean age: ~ 12 years). Further, Heleno et al. [3] found significantly reduced postural sway for the OLS and larger reach distances for the YBT-LQ in young male soccer players (mean age: ~ 15 years) after five weeks of BT (3 sessions/week).

Despite the gain in knowledge about positive effects of BT on measures of static and dynamic balance in adolescents, it remains unclear by which training frequency the greatest effects are achieved. In the previously mentioned studies [2, 3], BT frequencies varied from two to three times per week. However, a direct comparison of the reported effects is not possible due to differences in the used methodological approach (i.e., balance tests/outcomes, training durations/volumes etc.).

Therefore, the present study aimed to compare the effectiveness of a 6-week BT program with equal volume but different frequencies (i.e., 2 times/week with 30 min per session versus 3 times/week with 20 min per session) on measures of static and dynamic balance in healthy male adolescents. First, it was hypothesized that both training programs will result in balance improvements. Second, it was expected that a more compared to a less frequent exposure to BT stimuli will lead to greater improvements.

## Main text

### Methods

#### Participants

Thirty-two male adolescents (age: 15.2 ± 0.9 years; body height: 177.3 ± 9.8 cm, body mass: 66.4 ± 12.0 kg; body mass index: 20.9 ± 2.3 kg/m^2^) participated in this study and were randomly assigned to the BT-2x/wk group (i.e., 2 sessions per week, 30 min each) or the BT-3x/wk group (i.e., 3 sessions per week, 20 min each). All participants were healthy and free of any neurological or musculoskeletal impairment. None of the participants had prior experience with the balance assessments. Written informed consent and subject’s assent were obtained from all participants before the start of the study. In addition, parent’s approval was obtained for minors.

#### Balance assessment

**Fig. 1:**
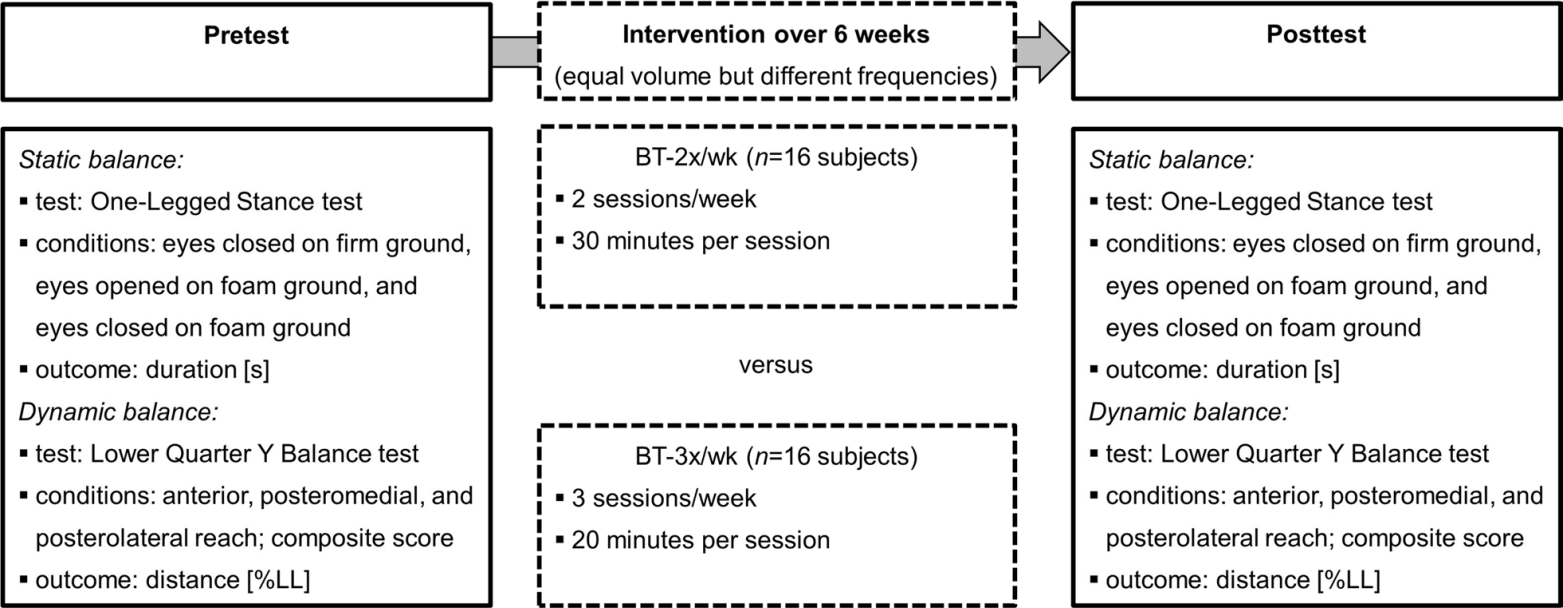
Schematic description of the study design. *BT* balance training, *LL* leg length

Balance assessment was conducted in a gym hall by the same skilled assessors (graduated sport scientists) before and after the six week training period. The timed OLS test was used to assess static balance performance (Fig. 1). Participants were asked to stand without shoes on their non-dominant leg (determined by self-report) for as long as possible but for a maximum of 60 s. The OLS was conducted with (a) eyes closed on firm ground (EC-FI), (b) eyes opened on foam (i.e., AIREX balance pad) ground (EO-FO), and (c) eyes closed on foam ground (EC-FO). After a practice trial, one data-collection trial was executed, and the maximal stance time (s) during each condition was used for further analysis. The timed OLS test is a valid (concurrent and discriminative) and reliable (moderate to excellent) test of balance performance in youth [4, 5].

The YBT-LQ test was used to measure dynamic balance performance (Fig. 1). Participants were instructed to stand barefoot with their non-dominant leg on the central footplate and to reach with their dominant leg as far as possible in the anterior (AT), posteromedial (PM), and posterolateral (PL) direction. Following three practice trials, three data-collection trials were performed and the absolute maximal reach distance (cm) per reach direction was noted. Afterwards, these values were normalized by dividing the absolute maximal reach distance by leg length (LL) and then multiplying by 100. Further, the normalized (% LL) composite score (CS) was calculated as the sum of the absolute maximal reach distance per reach direction divided by three times LL and then multiplied by 100. Participant’s LL was determined from the anterior superior iliac spine to the most distal aspect of the medial malleolus [6]. The YBT-LQ test is a valid (concurrent, discriminative, and predictive) and reliable (moderate to excellent) tool to assess balance performance in youth [7–9].

#### Balance training

Balance training was conducted at the school gym and supervised by the respective physical education teacher and two graduated students. Both groups trained balance for six weeks with an equal volume but different training frequencies (i.e., 2 × 30 min per week or 3 × 20 min per week) (Fig. 1). Each session included five balance exercises that were performed twice per leg. Training progression was achieved by using unstable training devices, removing visual information, or changing exercise durations/repetitions (Table 1).

**Table 1:** Description of the 6-week balance training program

Exercises	Week 1	Week 2	Week 3	Week 4	Week 5	Week 6
Unipedal stance on a balance pad^1^	2 × 30/45 s (EO)	2 × 30/45 s (EO)	2 × 30/45 s (EO)	2 × 30/45 s (EC)	2 × 30/45 s (EC)	2 × 30/45 s (EC)
Unipedal stance on a wobble board^2^	2 × 30/45 s (level 1–2)	2 × 30/45 s (level 1–2)	2 × 30/45 s (level 3–4)	2 × 30/45 s (level 3–4)	2 × 30/45 s (level 5–6)	2 × 30/45 s (level 5–6)
Unipedal weight shifting	2 × 30/45 s (a-p, EO-FO)	2 × 30/45 s (m-l, EO-FO)	2 × 30/45 s (a-p, EC-FI)	2 × 30/45 s (m-l, EC-FI)	2 × 30/45 s (a-p, EC-FO)	2 × 30/45 s (m-l, EC-FO)
Unipedal vertical jump-landings	2 × 10/15 reps (EO-FO)	2 × 10/15 reps (EO-FO)	2 × 10/15 reps (EC-FI)	2 × 10/15 reps (EC-FI)	2 × 10/15 reps (EC-FO)	2 × 10/15 reps (EC-FO)
Unipedal horizontal jump-landings	2 × 10/15 reps (EO-FO)	2 × 10/15 reps (EO-FO)	2 × 10/15 reps (EC-FI)	2 × 10/15 reps (EC-FI)	2 × 10/15 reps (EC-FO)	2 × 10/15 reps (EC-FO)

Note, the first duration (i.e., 30 s) and number of repetitions (i.e., 10 reps) refers to BT-3x/wk and the second duration (45 s) and number of repetitions (i.e., 15 reps) refers to BT-2x/wk. ^1^AIREX^®^ balance pad; ^2^ARTZT^®^ vitality Wobblesmart board; a-p = anterior–posterior; *EC* eyes closed, *EO* eyes opened, *FI* firm ground, *FO* foam ground, *m-l* medio-lateral

#### Statistical analyses

Descriptive data are reported as group means ± standard deviations. A 2 (group: BT-2x/wk, BT-3x/wk) × 2 (test: pretest, posttest) repeated measures analysis of variance (ANOVA) was conducted to detect training-related group differences. Additionally, effect size (*η*_p_^2^) was calculated and reported as small (0.02 ≤ *η*_p_^2^ ≤ 0.12), medium (0.13 ≤ *η*_p_^2^ ≤ 0.25), or large (*η*_p_^2^ ≥ 0.26) [10]. All statistical analyses were conducted using Statistical Package for Social Sciences version 27.0 and the α-value was a priori set at *p* < 0.05.

### Results

#### Static balance performance

For all stance conditions, the repeated measures ANOVA showed significant medium- to large-sized main effects of test (Table 2). This indicates improvements in stance duration independent of the applied training frequency. The main effect of group and the group × test interaction did not reach the level of significance.

**Table 2:** Effects of balance training frequency on measures of static and dynamic balance in adolescents

Measure	BT-2x/wk (*n* = 16)		BT-3x/wk (*n* = 16)		*p*-value (*η*_p_^2^)		
	Pretest	Posttest	Pretest	Posttest	Group	Test	Group × Test
*OLS*							
OLS time; EC-FI [s]	38.4 ± 18.6	49.2 ± 18.7	24.5 ± 24.7	41.7 ± 22.3	0.103 (0.09)	0.001 (0.29)	0.426 (0.02)
OLS time; EO-FO [s]	48.7 ± 18.7	60.0 ± 0.2	53.6 ± 14.0	56.8 ± 7.1	0.800 (0.01)	0.016 (0.18)	0.166 (0.06)
OLS time; EC-FO [s]	4.7 ± 2.9	9.6 ± 12.6	4.7 ± 2.5	13.7 ± 15.9	0.446 (0.02)	0.008 (0.21)	0.401 (0.02)
*YBT-LQ*							
YBT-LQ: AT reach [% LL]	67.9 ± 5.4	70.3 ± 5.1	65.6 ± 4.8	69.5 ± 5.4	0.388 (0.03)	< 0.001 (0.52)	0.175 (0.06)
YBT-LQ: PM reach [% LL]	107.3 ± 4.1	111.9 ± 4.1	106.0 ± 5.0	112.7 ± 5.8	0.858 (0.01)	< 0.001 (0.69)	0.134 (0.07)
YBT-LQ: PL reach [% LL]	101.2 ± 7.1	108.1 ± 4.5	100.8 ± 7.1	106.8 ± 5.6	0.676 (0.01)	< 0.001 (0.63)	0.595 (0.01)
YBT-LQ: CS [% LL]	92.1 ± 3.9	96.8 ± 3.6	90.8 ± 4.4	96.3 ± 4.3	0.521 (0.01)	< 0.001 (0.80)	0.349 (0.03)

Data represent means ± standard deviations. Values are *p*-values and effect sizes (*η*_p_^2^) in brackets with 0.02 ≤ *η*_p_^2^ ≤ 0.12 indicating small, 0.13 ≤ *η*_p_^2^ ≤ 0.25 indicating medium, and *η*_p_^2^ ≥ 0.26 indicating large effects. *AT* anterior; *CS* composite score; *EC* eyes closed; *EO* eyes opened; *FI* firm ground; *FO* foam ground; *LL* leg length; *OLS* One-Legged Stance test; *PL* posterolateral; *PM* posteromedial; *YBT-LQ* Lower Quarter Y Balance test

#### Dynamic balance performance

Irrespective of reach direction, the repeated measures ANOVA yielded significant large-sized main effects of test (Table 2). Again, this implies enhancements in reach distance independent of the used training frequency. Neither the main effect of group nor the group × test interaction reached the level of significance.

### Discussion

In line with our first hypothesis stating that BT will result in balance improvements, both groups significantly increased (medium to large effect sizes) their static (OLS, standing duration) and dynamic (YBT-LQ, reach distance) balance performance. These findings are in accordance with those from previous studies that investigated the effect of BT in healthy male adolescents [2, 3] and indicate that BT is an effective training regimen to enhance static and dynamic balance in youth. Second, it was expected that a more compared to a less frequent exposure to BT stimuli will lead to greater improvements. Contrary to this hypothesis, no significant group by test interactions were detected neither for static nor for dynamic balance performance. This indicates that three compared to two training sessions per week did not result in group-specific balance improvements. This result is consistent with the findings of the systematic review with meta-analysis conducted by Gebel et al. [1]. The authors performed indirect (between-study) comparisons and reported medium- and large-sized improvements for both approaches (i.e., BT conducted twice and three times per week). However, they did not detect significant differences between the two training frequencies.

What are possible reasons that the effectiveness of BT was not affected by differences in training frequency? One reason might be that the training duration of six weeks may have been too short to detect significantly greater adaptations as a function of training frequency. Thus, further studies should investigate whether the supposed additional value of a more frequent exposure to BT stimuli becomes apparent after several months of BT. Another explanation could be that the advantage of a more frequent exposure may have been compensated by the disadvantage of a shorter period for ongoing recovery and consolidation processes [11]. Subsequent studies should therefore investigate whether a greater frequency of BT stimuli (e.g., 4 times/week) will even cause negative effects.

### Conclusion

The present study investigated the effects of a 6-week BT with equal training volume but different training frequencies (i.e., 2 × 30 min per week or 3 × 20 min per week) on parameters of static and dynamic balance in healthy male adolescents. We observed significant improvements in static (i.e., increased stance durations using the OLS test) and dynamic (i.e., increased reach distances using the YBT-LQ test) balance performance. However, the enhancements were not differentially affected by the applied training frequency. These results imply that BT is an effective training regimen in healthy male adolescents but a more compared to a less frequent exposure to BT stimuli does not seem to provide additional effects.

## Limitations

- Only male adolescents were investigated, which limits the transferability of the present results to female adolescents.
- Frequently used field tests (i.e., OLS, YBT-LQ) but no instrumented biomechanical testing (e.g., center of pressure displacements using a force platform) were applied.
- The observed effects refer to a BT of several weeks and cannot be transferred to a BT of several months.
- Training-related changes on a behavioural level (i.e., stance duration and reach distance) but no adaptations on a neuromuscular level (e.g., muscle activity) were investigated.

## Data Availability

The data generated and analyzed during the present study are not publicly available due to ethical restrictions but are available from the corresponding author upon reasonable request.

## Abbreviations

ANOVA: Analysis of variance
AT: Anterior
CS: Composite score
EC: Eyes closed
EO: Eyes opened
FI: Firm ground
FO: Foam ground
LL: Leg length
OLS: One-Legged Stance test
PL: Posterolateral
PM: Posteromedial
YBT-LQ: Lower Quarter Y Balance test
